# NF-κB activation is a turn on for vaccinia virus phosphoprotein A49 to turn off NF-κB activation

**DOI:** 10.1073/pnas.1813504116

**Published:** 2019-02-28

**Authors:** Sarah Neidel, Hongwei Ren, Alice A. Torres, Geoffrey L. Smith

**Affiliations:** ^a^Department of Pathology, University of Cambridge, Cambridge CB2 1QP, United Kingdom

**Keywords:** vaccinia virus, nuclear factor kappa B, virus immune evasion, innate immunity

## Abstract

Vaccinia virus protein A49 inhibits NF-κB activation by molecular mimicry and has a motif near the N terminus that is conserved in IκBα, β-catenin, HIV Vpu, and some other proteins. This motif contains two serines, and for IκBα and β-catenin, phosphorylation of these serines enables recognition by the E3 ubiquitin ligase β-TrCP. Binding of IκBα and β-catenin by β-TrCP causes their ubiquitylation and thereafter proteasome-mediated degradation. In contrast, HIV Vpu and VACV A49 are not degraded. This paper shows that A49 is phosphorylated at serine 7 but not serine 12 and that this is necessary and sufficient for binding β-TrCP and antagonism of NF-κB. Phosphorylation of A49 S7 occurs when NF-κB signaling is activated by addition of IL-1β or overexpression of TRAF6 or IKKβ, the kinase needed for IκBα phosphorylation. Thus, A49 shows beautiful biological regulation, for it becomes an NF-κB antagonist upon activation of NF-κB signaling. The virulence of viruses expressing mutant A49 proteins or lacking A49 (vΔA49) was tested. vΔA49 was attenuated compared with WT, but viruses expressing A49 that cannot bind β-TrCP or bind β-TrCP constitutively had intermediate virulence. So A49 promotes virulence by inhibiting NF-κB activation and by another mechanism independent of S7 phosphorylation and NF-κB antagonism. Last, a virus lacking A49 was more immunogenic than the WT virus.

## Significance

Vaccinia virus (VACV) encodes many proteins that inhibit innate immunity. For instance, protein A49 inhibits NF-κB activation by binding to β-TrCP. Here we show that A49 is phosphorylated on serine 7 and that this is necessary for binding β-TrCP and inhibition of NF-κB activation. Further, this phosphorylation occurs when the NF-κB pathway is stimulated and the kinase IKKβ is activated. Thus, A49 shows beautiful biological regulation, for activation of the pathway also activates the virus inhibitor of the pathway. The significance is seen in vivo, since VACVs expressing A49 S7A or S7E are less virulent than wild-type virus but more virulent than a virus lacking A49.

The *Poxviridae* includes the *Orthopoxvirus* genus of which the most studied species is *Vaccinia virus* (VACV) (1). Like other poxviruses, VACV has a large, complex virion, a cytoplasmic site of replication (2), and a dsDNA genome encoding about 200 genes (3). Orthopoxvirus genomes have a highly conserved central region and more variable termini (4). The central region encodes proteins needed for replication, while the terminal regions encode proteins that affect virus virulence, host range, and immunomodulation. Many of the latter proteins are dispensable for replication in cell culture but suppress innate immunity and are important in vivo (5). These immunomodulatory proteins are numerous, and many target the same signaling pathway. For instance, VACV encodes at least 10 proteins that inhibit activation of NF-κB (5, 6). This article concerns one NF-κB inhibitor, protein A49.

A49 is a small intracellular protein that contributes to virus virulence (7). A49 has a B cell lymphoma (Bcl)-2-like fold (8) and is one of 11 Bcl-2-like proteins encoded by VACV. Some of these mimic cellular Bcl-2 family proteins with antiapoptotic activity. For instance, proteins N1 (9–11) and F1 (12) inhibit apoptosis (10, 11, 13, 14). However, VACV Bcl-2 proteins B14, A52 (15), and A46 (16, 17) do not inhibit apoptosis but inhibit other innate immune signaling pathways (18–22). A49 most closely resembles myxoma virus protein M11, an antiapoptotic protein (23), but does not bind the cellular proapoptotic Bcl-2 proteins bound by M11 (8).

A49 inhibits activation of the IFN-β promoter (7) by blocking NF-κB signaling via molecular mimicry (7). Near its N terminus, A49 contains two serines that are conserved in several proteins, such as IκBα and β-catenin (24), and as viral proteins HIV Vpu (25, 26) and rotavirus nonstructural protein 1 (NSP1) (27). For IκBα, these serines are phosphorylated by IKKβ that is activated during NF-κB signaling. Once phosphorylated, IκBα is recognized by the E3 ubiquitin ligase, beta-transducin repeat-containing protein (β-TrCP) (24), which ubiquitylates upstream lysine residues, leading to proteasomal degradation of IκBα (28). This releases the NF-κB subunits p65 and p50 into the nucleus.

A49 binds to β-TrCP and prevents ubiquitylation of phosphorylated (p)-IκBα and thereby stabilizes it (7). A49 also stabilizes another β-TrCP substrate, β-catenin, leading to activation of the wnt signaling pathway (29). The interaction of A49 with β-TrCP requires either or both of serines 7 and 12, for mutation of both residues to alanine prevented binding to β-TrCP and NF-κB antagonism (7). In contrast, mutation to glutamic acid enhanced binding to β-TrCP and increased NF-κB antagonism, suggesting A49 needs phosphorylation to be an NF-κB inhibitor.

Here A49 is shown to be phosphorylated on S7 but not S12, and this is necessary and sufficient for binding to β-TrCP and antagonism of NF-κB activation. Further, A49 is phosphorylated when NF-κB signaling is activated. Therefore, A49 functions to inhibit NF-κB signaling conditionally, when this signaling pathway is activated. VACVs expressing mutant A49 unable to bind β-TrCP and antagonize NF-κB signaling or expressing A49 binding β-TrCP constitutively each had intermediate virulence between WT virus and a virus lacking the *A49R* gene (vΔA49). This indicates that A49 promotes virulence by inhibiting NF-κB activation and another function. Last, a VACV lacking A49 was more immunogenic than WT virus and provided better protection against VACV challenge.

## Results

### A49 Is Phosphorylated.

**Fig. 1.**
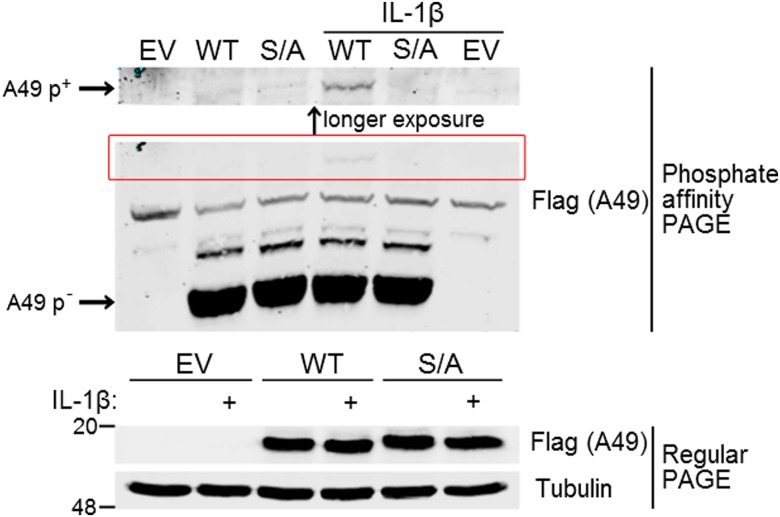
A49 is phosphorylated upon stimulation of NF-κB signaling. HeLa cells were transfected with codon-optimized, FLAG-tagged wildtype A49 (WT) or A49 mutant S7/12A (S/A) or an EV control. After 24 h, cells were stimulated for 30 min with 50 ng/mL IL-1β or left unstimulated before lysis in 50 mM Tris (pH 7.4), 250 mM NaCl, 0.5% Nonidet P-40, supplemented with protease inhibitors and NaF. Cleared lysates were mixed with SDS loading buffer, boiled, and analyzed immediately by phosphate-affinity PAGE to separate phosphorylated (p^+^) from unphosphorylated (p^−^) A49 and regular PAGE, followed by immunoblotting.

The cellular proteins β-catenin and IκBα are phosphorylated to enable efficient binding to β-TrCP, and the structure of β-TrCP bound to p-β-catenin shows extensive interactions between the phosphate groups of β-catenin and the β-TrCP binding pocket (30). To examine if A49 is also phosphorylated, a Phos-tag was introduced into polyacrylamide gels as described previously (31). Phosphorylated proteins bind the Phos-tag and so migrate more slowly during gel electrophoresis. Plasmids expressing codon-optimized, FLAG-tagged WT A49 or in which serines 7 and 12 are changed to alanines (S7/12A) (7) were transfected into HeLa cells in parallel with an empty vector (EV). These cells were left untreated or were treated with IL-1β before harvesting and analysis by phosphate-affinity PAGE and regular SDS/PAGE and immunoblotting (Fig. 1). The levels of WT and mutant A49 detected by the anti-FLAG antibody were similar with or without IL-1β stimulation (Fig. 1, *Bottom*). However, the phosphate-affinity gel showed a slower migrating band that was present with WT A49 protein but not S7/12A and only after IL-1β stimulation (Fig. 1, *Top*). Compared with the nonphosphorylated A49 protein, the phosphorylated (p)-A49 band is weak, suggesting only a small fraction of A49 is phosphorylated or that, in the absence of a binding partner, it is dephosphorylated.

**Fig. 2.**
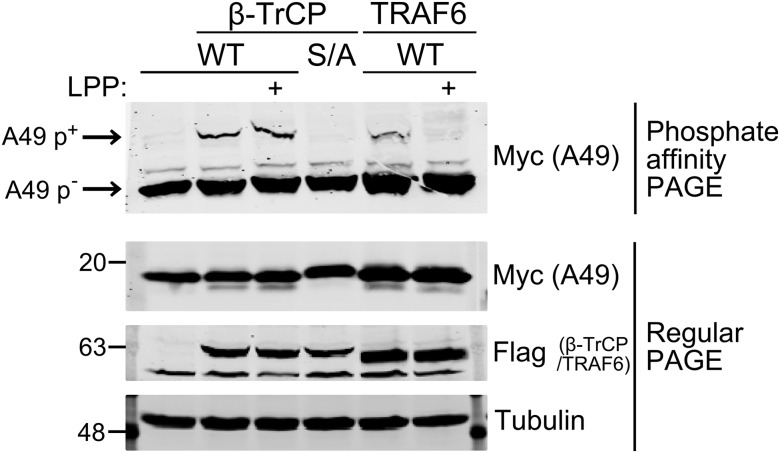
Phosphorylated A49 is protected from dephosphorylation by β-TrCP. HEK-293T cells were transfected with codon-optimized, myc-tagged wildtype A49 (WT) or A49 mutant S7/12A (S/A) and FLAG-tagged β-TrCP or TRAF6. Cells were lysed in Phos-tag gel lysis buffer supplemented with protease inhibitors and NaF and treated with lambda protein phosphatase (LPP) or not, as indicated. Samples were then mixed with SDS loading buffer, boiled, and analyzed immediately by phosphate-affinity PAGE to separate phosphorylated (p^+^) from unphosphorylated (p^−^) A49 and regular PAGE, followed by immunoblotting.

To confirm this extra band is p-A49, samples were treated with phosphatase (LPP). In this case, myc-tagged WT and S7/12A mutant A49 were expressed in HEK-293T cells, together with FLAG-tagged β-TrCP. In parallel, WT A49 was transfected either alone or with FLAG-tagged TRAF6 to activate NF-κB signaling. SDS/PAGE showed expression of FLAG-tagged β-TrCP or TRAF6 and myc-tagged A49 proteins (Fig. 2, *Bottom*). In the phosphate-affinity gel, there was a slower migrating A49 band, seen only when the pathway was activated by TRAF6 (compare lanes 1 and 5), and treatment with LPP caused loss of this band (lane 6). When β-TrCP was overexpressed, p-A49 was seen without pathway stimulation and was resistant to dephosphorylation, suggesting A49–β-TrCP interaction is stable and protects p-A49 from dephosphorylation (lanes 2 and 3). The stabilization induced by β-TrCP overexpression may explain why p-A49 was detectable without the addition of IL-1β or expression of TRAF6 to activate the pathway.

### A49 Is Phosphorylated on Only Serine 7.

**Fig. 3.**
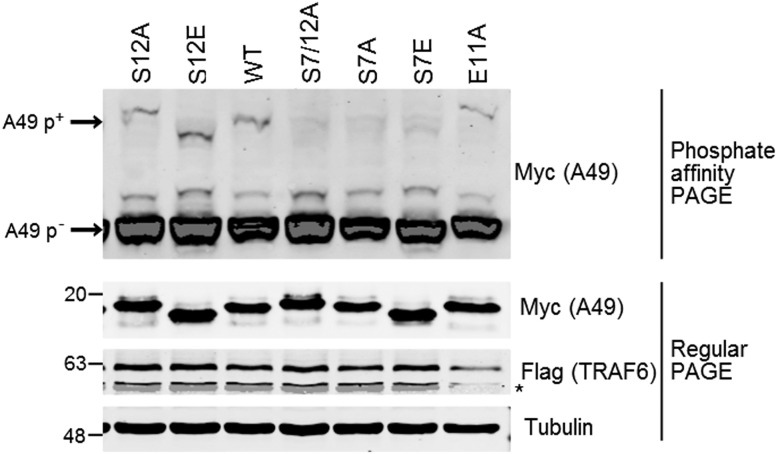
A49 is phosphorylated on serine 7 but not serine 12. HEK-293T cells were transfected with codon-optimized, myc-tagged wildtype A49 (WT) or A49 mutants and FLAG-tagged TRAF6. Cells were lysed in IP buffer (*Materials and Methods*), and cleared cell lysates were mixed with SDS loading buffer, boiled, and analyzed immediately by phosphate-affinity PAGE to separate phosphorylated (p^+^) from unphosphorylated (p^−^) A49 and regular PAGE. The star marks a nonspecific signal detected by the anti-FLAG antibody.

To investigate which serines are phosphorylated, additional mutant A49 proteins were constructed. These comprised S7A, S12A, and also S7E, S12E, and E11A. S7E and S12E were included because, previously, a mutant A49 with both these serines mutated to glutamic acid (S7/12E) bound to β-TrCP strongly and was a stronger inhibitor of NF-κB activation (7). E11A was included because, compared with IκBα, A49 contains an extra aa before S12. Since this residue is glutamic acid, a phosphomimetic, this might substitute for a phosphoserine at S12. WT and mutant myc-tagged A49 alleles were transfected into cells together with FLAG-tagged TRAF6, and cell lysates were analyzed as above (Fig. 3). SDS/PAGE showed the expression and slightly differing electrophoretic mobility of these mutants (Fig. 3, *Bottom*). The phosphoaffinity gel showed a p-A49 band with WT, S12A, S12E, and E11A but not with S7/12A, S7A, or S7E (Fig. 3, *Top*). Therefore, S7 but not S12 is phosphorylated.

### Phosphorylation of A49 Is Needed for Binding β-TrCP and Inhibition of NF-κB Activation.

**Fig. 4.**
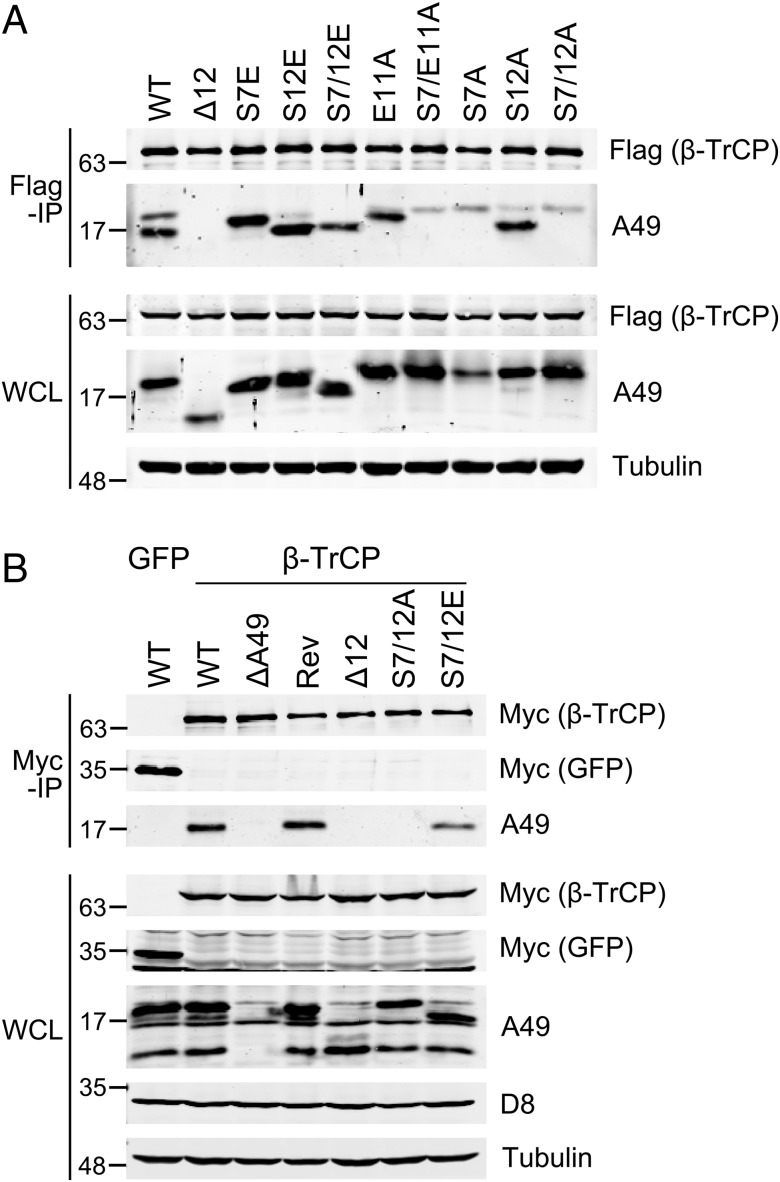
p-A49 coimmunoprecipitates with β-TrCP. (*A*) HEK-293T cells were transfected with FLAG-tagged β-TrCP and untagged, codon-optimized wildtype A49 (WT) or its mutants. Cells were lysed after 24 h in IP buffer supplemented with protease and phosphatase inhibitors, and cleared lysates were incubated for 16 h with a FLAG-affinity gel. Bound proteins were washed with lysis buffer and eluted with SDS loading buffer before they were analyzed by immunoblot, together with the whole cell lysate (WCL). (*B*) HEK-293T cells were transfected with plasmids expressing either myc-tagged GFP or myc-tagged β-TrCP. After 24 h, the cells were infected at 5 pfu/cell with the indicated VACVs for 16 h. Cells were then treated as in *A*, with the exception that a myc-affinity gel was used for immunoprecipitation.

The ability of these A49 mutants to bind β-TrCP and inhibit NF-κB signaling was tested. HEK-293T cells were transfected with FLAG-tagged β-TrCP and untagged, codon-optimized WT A49 or mutants. The FLAG-tagged β-TrCP was immunoprecipitated from cell lysates and analyzed by immunoblotting with anti-FLAG and anti-A49 polyclonal antibody (7) (Fig. 4*A*). Note the slightly different electrophoretic mobility of these untagged A49 proteins compared with that of the myc-tagged A49 proteins shown in Fig. 3. All mutants with S7 or S7E were immunoprecipitated by β-TrCP, and all mutants lacking S7 (or S7E) were not or only very poorly. Thus, S7 phosphorylation, or S7E at this position, is needed for binding β-TrCP. Similar analyses in which myc-tagged β-TrCP or myc-tagged GFP was transfected into cells that subsequently were infected with WT or A49 mutant viruses also showed that WT A49 or A49 S7/12E was coprecipitated with β-TrCP, whereas A49 Δ12 or A49 S7/12A was not (Fig. 4*B*). Equal infection and equal loading were shown using antibody to VACV protein D8 and α-tubulin, respectively.

**Fig. 5.**
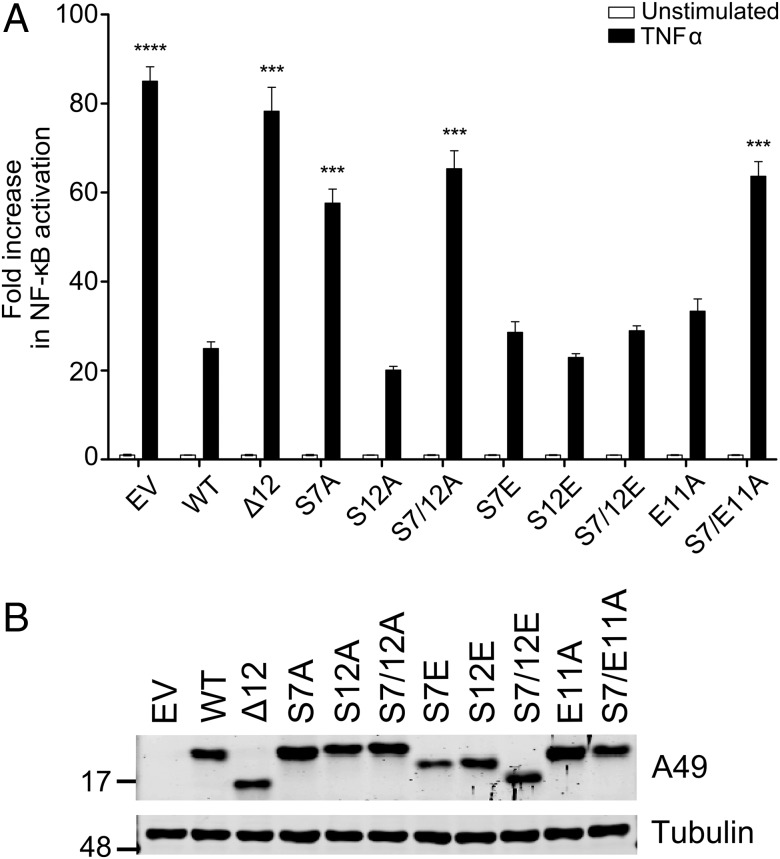
Mutation of serine 7 abolishes inhibition of NF-κB activation by A49. (*A*) HEK-293T cells were transfected with NF-κB-luc, TK-renilla, and plasmids encoding WT or mutant A49 proteins or EV. After 24 h cells were stimulated by the addition of 15 ng/mL TNFα for 8 h. Luciferase activity was measured in cell lysates. Triplicate samples were analyzed for each condition. Data are expressed as the mean fold induction of the firefly luciferase activity normalized to renilla values for the stimulated versus unstimulated samples. Data are then expressed as the EV value compared with the test sample ± SEM. ***P < 0.001; ****P < 0.0001. (*B*) Immunoblot showing the expression levels of A49 and α-tubulin analyzed in *A*.

The ability of A49 mutants to block NF-κB activation was measured using a NF-κB responsive promoter linked to luciferase, as described previously (7). WT A49 and mutants S12A, S7E, S12E, S7/12E, and E11A all inhibited NF-κB activation, but S7A, S7/12A, S7/E11A, and a mutant lacking the first 12 aa (Δ12) did not (Fig. 5*A*). Immunoblotting of cell lysates with anti-A49 antibody and anti-α-tubulin confirmed equivalent expression of these proteins and equal loading (Fig. 5*B*). In summary, the phosphorylation of A49 occurs at S7, and this is critical for A49 to bind β-TrCP and antagonize NF-κB signaling.

### IKKβ Can Phosphorylate A49.

**Fig. 6.**
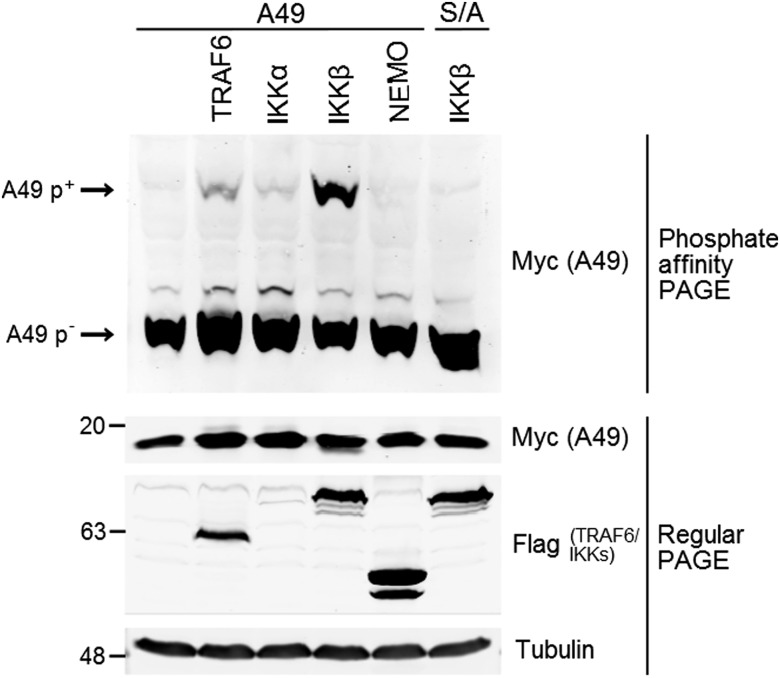
A49 is phosphorylated after activation of NF-κB signaling at or downstream of the IKK complex. HEK-293T cells were transfected with codon-optimized, myc-tagged A49 alone or together with FLAG-tagged TRAF6, IKKα, IKKβ, or NEMO. As a control, cells were cotransfected with the S7/12A mutant of A49 and IKKβ. Cells were lysed in Phos-tag gel buffer supplemented with NaF, and cleared lysates were mixed with SDS loading buffer, boiled, and stored at −80 °C before analysis by phosphate-affinity PAGE to separate phosphorylated (p^+^) from unphosphorylated (p^−^) A49 and regular PAGE.

Stimulation of NF-κB signaling via addition of IL-1β or overexpression of TRAF6 caused phosphorylation of A49 (Figs. 1 and 2). Both stimuli cause phosphorylation and activation of IKKβ, leading to phosphorylation of IκBα. Under these conditions, A49 was phosphorylated, suggesting that IKKβ might phosphorylate A49. To test this, cells were transfected with myc-tagged A49 together with FLAG-tagged TRAF6, IKKα, IKKβ, or NEMO, and cell lysates were analyzed by SDS/PAGE or phosphate-affinity PAGE (Fig. 6). SDS/PAGE confirmed expression of all these proteins, except for IKKα, which was barely detectable. Phosphoaffinity PAGE showed that expression of IKKβ induced strong phosphorylation of A49 and that TRAF6 was less effective. Trace amounts of p-A49 were also seen with low expression of IKKα, but no phosphorylation of A49 was observed when NEMO was expressed. In parallel, substitution of WT A49 for the S7/12A mutant confirmed lack of A49 phosphorylation even when IKKβ was expressed. In summary, IKKβ can induce phosphorylation of A49.

### VACV Expressing Nonphosphorylated A49 Is Less Virulent than Wild-Type Virus.

**Fig. 7.**
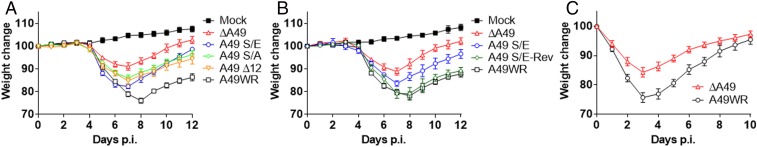
Virulence and immunogenicity of VACV A49 mutant viruses. (*A*) Female BALB/c mice (6–8 wk old, *n* = 5) were mock-infected or infected intranasally with 5 × 10^3^ pfu of the indicated viruses (10 µL/nostril), and their weight was monitored daily. Weight data are expressed as the percentage ± SEM of the mean weight of the same group of animals on day 0. Statistical analyses compared mutant viruses with one another and with vA49 and vΔA49 viruses using two-way ANOVA. (*B*) As in *A*, except using VACV WT (A49WR), A49 S7/12E (A49 S/E), ΔA49, and A49 S7/12E-rev (A49 S/E-rev). A49WR and A49 S/E-rev were not statistically different. (*C*) Mice immunized with VACV WT or ΔA49 were challenged i.n. with 1 × 10^7^ pfu of wild-type VACV WR at 42 d postinfection. Mice were monitored as in *A*. Statistical analyses using two-way ANOVA showed the difference between these two groups was significant; *P* = 0.012. Data shown in *A* and *C* are from one representative experiment out of two, and those in *B* are from a single experiment.

A virus mutant lacking gene *A49R*, vΔA49, was attenuated in a murine intranasal model (7). To determine if the ability of A49 to bind β-TrCP and thus inhibit NF-κB signaling is the only reason for attenuation, recombinant VACVs bearing A49 S7/12A or S7/12E or lacking the first 12 aa (Δ12) were constructed, and the virulence of these viruses was tested by measuring weight change after infection (Fig. 7*A*). Notably, all these mutant viruses had intermediate virulence that was statistically different from both WT and deletion mutant. For the A49 S7/12E mutant virus, the mutant *A49R* gene was replaced with the WT gene (A49 S7/12E-rev). The virulence of this virus was the same as WT (Fig. 7*B*), confirming that the intermediate virulence of the S7/12E virus was due to A49 mutation. For the other mutant viruses (S71/12A and Δ12), additional revertant viruses were not made; instead, complete genome sequencing confirmed that there were no other mutations compared with WT. Collectively, these analyses showed that A49 binding to β-TrCP and thereby inhibiting NF-κB is important for virulence. However, because viruses with mutant A49 proteins unable to inhibit NF-κB were more virulent than vΔA49, A49 has an additional function that contributes to virulence. Surprisingly, the virulence of the A49 S7/12E mutant virus was also intermediate. This A49 protein binds β-TrCP and inhibits NF-κB signaling at least as efficiently as WT A49 (7), yet the virus has reduced virulence. A possible explanation is that binding β-TrCP constitutively, whether or not the NF-κB pathway is activated, might prevent nonphosphorylated A49 unbound by β-TrCP from having another function. This would be consistent with the observation that a virus expressing A49 that cannot bind β-TrCP has intermediate virulence.

### Deletion of A49 Increases VACV Immunogenicity.

In several cases loss of one immunomodulator from VACV increases virus immunogenicity despite virus attenuation. To investigate if this was true for A49, mice immunized with vA49WR or vΔA49 were challenged intranasally 42 d later with WT VACV strain Western Reserve (WR). Animals immunized with vΔA49 had significantly lower weight loss than those with WT virus (Fig. 7*C*). The viruses with mutated A49 proteins were intermediate between WT and vΔA49, but these small differences did not reach statistical significance (*SI Appendix*, Fig. S1).

## Discussion

This article reports that VACV protein A49 is phosphorylated on serine 7 when the NF-κB signaling pathway is turned on. The addition of IL-1β or overexpression of TRAF6 or IKKβ caused A49 phosphorylation, and this enabled p-A49 binding to β-TrCP. Sequestration of β-TrCP by p-A49 prevents β-TrCP from recognizing other substrates such as p-IκBα (7) or p-β-catenin (29), so these substrates remain stable. β-TrCP has several other substrates, for instance, CDC25 (32), that might also be affected. For β-catenin, its stabilization leads to activation of the wnt signaling pathway (29). For p-IκBα, its stabilization causes retention of cytoplasmic NF-κB p65 and p50 and no transcription from NF-κB–dependent promoters. Since the ability of A49 to bind to β-TrCP is dependent on phosphorylation and phosphorylation can be achieved by IKKβ activation, the activation of NF-κB signaling is a turn on for A49 to turn off NF-κB activation. This represents beautiful biological regulation, for without the need to inhibit NF-κB, A49 remains unbound to β-TrCP and free to undertake other functions.

In vivo evidence indicates that A49 has a second function. The virulence of VACV expressing mutant A49 that is not phosphorylated, no longer binds β-TrCP, and no longer inhibits NF-κB activation is intermediate between WT and vΔA49. If inhibiting NF-κB activation were the sole function of A49, a virus expressing A49 unable to be phosphorylated would likely have virulence equivalent to vΔA49. Analysis of VACV expressing A49 S7/12E provides further evidence for another A49 function. A49 S7/12E binds β-TrCP constitutively, but despite this, VACV expressing A49 S7/12E is attenuated compared with WT. This attenuation may arise from A49 being constitutively bound to β-TrCP and thus unable to bind to other substrates and mediate other functions. An alternative hypothesis, that transient early activation of NF-κB is beneficial for the virus, seems less likely because the virus expresses many other NF-κB inhibitors early after infection.

Possible other functions for A49 include bringing other substrates to β-TrCP for ubiquitylation and proteasomal degradation, rather as HIV Vpu induces degradation of CD4 (26). Such a function would be lost by mutation of A49 to prevent β-TrCP binding. Alternatively, A49 might bind to other substrates without inducing their degradation to inhibit or modify their function. Some rotavirus NSP1 proteins bind to β-TrCP and modify its function like A49, whereas other NSP1 proteins induce degradation of β-TrCP (as well as IRF3 and IRF7) (33). Further proteomic screens for additional A49 binding partners either in the presence of proteasomal inhibitors or using A49 mutants unable to bind β-TrCP are needed to identify other targets.

A49 is one of several virus proteins that contain the motif SXXXS and mimic cellular proteins with this motif. Such virus proteins include HIV Vpu (26), Epstein–Barr virus latent membrane protein 1 (34), and the NSP1 protein from some rotavirus strains (33). These viral proteins, like their cellular counterparts, are phosphorylated and thereafter are recognized by β-TrCP. Cellular proteins such as IκBα and β-catenin contain lysine residues just upstream of the SXXXS motif, and these are ubiquitylated by β-TrCP, leading to proteasomal degradation. This phosphorylation, ubiquitylation, and degradation are cellular mechanisms to control activation of the NF-κB and wnt signaling pathways. In contrast, the virus proteins lack upstream lysines and so are not ubiquitylated following phosphorylation and remain bound to β-TrCP, blocking its engagement with other substrates.

A49 differs from some other β-TrCP substrates in being phosphorylated on only one of the two serines (S7), and this is sufficient for binding β-TrCP. In contrast, phosphorylation of p105 on both serines 927 and 932 is needed for recognition by β-TrCP and subsequent degradation (35). Other β-TrCP substrates can be recognized without phosphorylation (36). In cases where phosphorylation is needed for binding to β-TrCP, the kinases mediating phosphorylation may differ. For instance, HIV Vpu is phosphorylated by casein kinase-2 (CK-2) at S52 and S56 (37), whereas IKKβ can induce phosphorylation of VACV A49, although other kinases may also do so. Exactly how p-A49 fits into the IκBα-binding pocket of β-TrCP is unknown, but phosphorylation of S7 is essential, and a phosphomimetic residue at S12 is tolerated. A49 differs from most cellular and viral proteins in containing an extra residue between the two serines and shares this property with p105. Removal or substitution of this residue does not prevent binding to β-TrCP.

Overexpression of β-TrCP increased p-A49 levels without activation of NF-κB signaling by upstream stimuli. This suggests that some A49 phosphorylation may occur without pathway activation due to constitutive low IKKβ activity or activity of other kinases and that p-A49 is stabilized by binding β-TrCP. Consistent with this, A49 is resistant to dephosphorylation when β-TrCP is overexpressed. In contrast, at endogenous β-TrCP levels, A49 may be dephosphorylated by phosphatases, so only a small fraction of A49 is phosphorylated in the steady state.

Last, A49 is one of more than 10 VACV proteins that all inhibit NF-κB activation and that, when deleted individually from VACV, cause an in vivo phenotype (5). Intuitively, attenuation from the loss of an inhibitor seems improbable if 10 other inhibitors remain. A possible explanation for this paradox is that these NF-κB inhibitors are multifunctional and the other functions are not compensated by the other NF-κB inhibitors. For instance, N1 inhibits NF-κB activation and apoptosis (10, 11), and evidence for more than one function for A49 is presented here.

In conclusion, VACV protein A49 is phosphorylated on serine 7, and this is required for binding to β-TrCP and inhibition of NF-κB signaling. Viruses expressing A49 unable to bind to β-TrCP or that bind β-TrCP constitutively have a virulence intermediate between WT and the A49 deletion mutant. Thus, A49 is a conditional inhibitor of NF-κB and is activated only when needed, namely, when NF-κB signaling is activated.

## Materials and Methods

### Cells.

HEK-293T and BSC-1 cells were grown in DMEM (Gibco) supplemented with 10% heat-treated (56 °C, 30 min) FBS (Harlan-Sera Lab), 100 U/mL penicillin, and 100 µg/mL streptomycin (P&S). HeLa cells and RK_13_ cells were grown in minimal essential medium (MEM, Gibco) supplemented with 10% FBS and P&S. HeLa cells were also supplemented with nonessential aa (Gibco).

### Viruses.

VACV strain WR and derivatives lacking the *A49R* gene, vΔA49, or in which the *A49R* gene was reinserted into vΔA49, vA49-Rev, were described (7). Additional VACVs expressing mutant A49 proteins were constructed by transient dominant selection (38) as described for vA49-Rev (7) using plasmids bearing mutant A49R alleles. Viruses were grown on RK_13_ cells and titrated by plaque assay on BSC-1 cells. For in vivo work, viruses were purified from cytoplasmic extracts of infected cells by sedimentation through a cushion of 36% (wt/vol) sucrose (39).

### Codon Optimization and Site-Directed Mutagenesis.

A version of gene *A49R* codon optimized for expression in human cells was purchased from Thermo Fisher Scientific. Mutations in either the WT *A49R* gene for expression from VACV or the codon-optimized *A49R* gene for expression in mammalian cells were made using the QuikChange Site-Directed Mutagenesis Kit (Agilent). Changes were confirmed by DNA sequencing.

### Reporter Gene Assays.

Reporter gene assays in HEK-293T cells using NF-κB–firefly luciferase and TK-renilla were done as described (7). These plasmids were transfected into cells together with plasmids expressing WT or mutant A49 proteins; the following day cells were stimulated with TNF or IL-1β (as indicated), and the levels of luciferase activity were determined. The fold induction of luciferase activity following pathway stimulation (normalized to renilla control) was calculated compared with unstimulated control. Statistical analyses compared the fold induction of the test sample to the stimulated WT.

### Genome Sequencing.

DNA was extracted from viruses that had been purified from infected cells by sedimentation through two sucrose cushions (36% wt/vol), and the complete genome sequence was determined by Illumina technology. The VACV-A49-S/A DNA gave 232,436 reads with a mean coverage density of 273 per nucleotide; the VACV-A49Δ12 had 294,154 reads with a mean coverage of 423, and the WT-VACV was also sequenced with 401,667 reads and a mean coverage of 556.

### Mobility Shift Detection of Phosphorylated Proteins.

HEK-293T cells were transfected and/or stimulated as described in the figure legends. Before harvest, cells were washed with PBS on ice, scraped into 1 mL PBS, collected by centrifugation at 500 × *g* for 5 min at 4 °C, and then lysed in 180 µL Phos-tag gel lysis buffer [20 mM Tris⋅HCl (pH 7.4), 140 mM NaCl, 10 mM CaCl_2_, 0.1% Triton X-100] with 10 mM NaF (New England Biolabs) if phosphatase treatment was performed. After 15 min on ice, the lysate was cleared by centrifugation at 18,845 × *g* for 20 min at 4 °C, and the supernatant was mixed with 6× loading buffer [final concentration of 50 mM Tris⋅HCl (pH 6.8), 2% SDS, 10% glycerol, 0.1% bromophenol blue] and either stored at −80 °C or analyzed immediately by SDS/PAGE.

### Phosphate-Affinity SDS/PAGE.

Polyacrylamide gels were prepared with Bis-Tris/HCl buffer at pH 6.8 (31) and the addition of Phos-tag acrylamide (40). Separating gels (10% acrylamide) were made with 357 mM Bis-Tris⋅HCl, 50 mM Phos-tag acrylamide, and 100 mM Zn(NO_3_)_2_, and stacking gels (4% acrylamide) were made with 357 mM Bis-Tris⋅HCl, without Phos-tag or Zn(NO_3_)_2_. For the running buffer, fresh 1 M sodium bisulfite (Sigma) solution was prepared, filtered and mixed with 20× 3-morpholinopropane-1-sulfonic acid (MOPS) buffer before electrophoresis to give final concentrations of 50 mM MOPS, 50 mM Tris, 0.1% (wt/vol) SDS, and 5 mM sodium bisulfite.

### Immunoblotting of Phos-tag Gels.

After electrophoresis the gel was soaked twice in NuPAGE transfer buffer (Thermo Scientific) supplemented with 10% (vol/vol) methanol and 5 mM sodium bisulfite for 15 min. Transfer and subsequent procedures were the same as described (7). The antibodies used for immunoblotting and the positions of molecular mass markers (kDa) are indicated on the figures.

### Phosphatase Treatment of Cell Lysates.

Cell lysates (39 µL), prepared as above, were mixed with 5 µL MnCl_2_ (New England Biolabs) and 5 µL 10× protein metallophosphatases (PMP) buffer (New England Biolabs). Samples that were lysed in the absence of NaF also received 1 µL lambda protein phosphatase (LPP; New England Biolabs). After incubation at 30 °C for 30 min, 6× SDS loading buffer was added, and samples were analyzed by Phos-tag PAGE.

### Immunoprecipitation.

Cells were lysed in immunoprecipitation (IP) buffer [20 mM Tris⋅HCl (pH 7.4), 150 mM NaCl, 10 mM CaCl2, 0.1% Nonidet P-40, 10% glycerol] supplemented with protease inhibitors (cOmplete EDTA-free; Roche) and phosphatase inhibitors (PhosSTOP; Roche) where indicated. Lysates were cleared by centrifugation and then incubated with anti-FLAG M2 affinity resin (Sigma) at 4 °C for 4 h or with anti-c-Myc agarose resin (Santa Cruz) at 4 °C overnight, washed four times with lysis buffer, mixed with protein loading buffer, and analyzed by SDS/PAGE.

### Antibodies and Cytokines.

The following antibodies were used: rabbit polyclonal anti-A49 (diluted 1:1,000) (7), mouse monoclonal AB1.1 against VACV protein D8 (diluted 1:1,000) (41), rabbit polyclonal anti-FLAG (diluted 1:1,000) (F7425; Sigma-Aldrich), mouse monoclonal anti-myc (diluted 1:1,000) (9B11; Cell-Signaling), and mouse monoclonal anti-α-tubulin (diluted 1:1,000) (DM1A; Millipore). TNF-α and IL-1β were purchased from Peprotech.

### In Vivo Experiments in Murine Models.

VACV was purified from cytoplasmic extracts of infected cells by two rounds of sedimentation through 36% (wt/vol) sucrose at 32,900 × *g* for 80 min. Virus was resuspended in 10 mM Tris⋅HCl (pH 9) and stored at −80 °C. Virus used for infections was diluted in PBS containing 1% BSA and the titer of the diluted virus that was used to infect mice was determined by plaque assay on the day of infection. Female BALB/c mice (*n* = 5, 6–8 wk old) were anesthetized and inoculated intranasally (i.n.) in both nostrils with 5 × 10^3^ pfu, and the body weight was measured daily thereafter (42). For the challenge experiments, mice that had been immunized i.n. were challenged i.n. 6 wk later with 1 × 10^7^ pfu of wild-type VACV WR. Mice were monitored daily to record body weight.

### Statistical Analysis.

Data were analyzed using an unpaired Student’s *t* test, with Welch’s correction where appropriate, or a two-way ANOVA test where appropriate using the GraphPad Prism statistical software (GraphPad Software). Statistical significance is expressed as follows: *P < 0.05, **P < 0.01, ***P < 0.001. Data are representative of at least two independent experiments.

### Ethics Statement.

This work was conducted under license PPL 70/8524 from the UK Home Office according to the Animals (Scientific Procedures) Act 1986, with approval from the University of Cambridge Ethical Review Board.

## Supplementary Material

Supplementary File
