# Supplementary material for: NF-κB activation is a turn on for vaccinia virus phosphoprotein A49 to turn off NF-κB activation
Source: Proc Natl Acad Sci U S A. 2019 Feb 28;116(12):5699–704. doi: 10.1073/pnas.1813504116 (PMC6431142; doi:10.1073/pnas.1813504116)
Supplement: Supplementary File [file pnas.1813504116.sapp.pdf]

Supplementary Figure 1. Female Balb/c mice (6-8 weeks old, n=5) were immunised intranasally with the indicated viruses at 5×10^3 p.f.u. and were challenged intranasally with 1×10^7 p.f.u. of wild-type VACV WR at 42 d p.i. The weight of each mouse was monitored daily after challenge. Weight data are expressed as the percentage ± SEM of the mean weight of the same group of animals on the day of challenge. Statistical analyses compared each mutant virus with each other and with vA49 and vΔA49 viruses using 2-way ANOVA. The differences between each mutant and ΔA49, A49WR, or each other, were not significant. Data shown are from one representative experiment out of two.

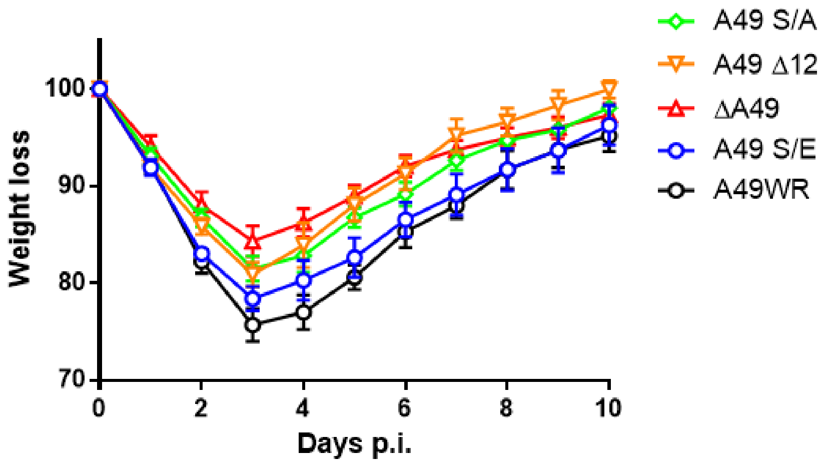

www.pnas.org/cgi/doi/10.1073/pnas.1813504116
